# Complementing sequence-derived features with structural information extracted from fragment libraries for protein structure prediction

**DOI:** 10.1186/s12859-021-04258-6

**Published:** 2021-06-28

**Authors:** Siyuan Liu, Tong Wang, Qijiang Xu, Bin Shao, Jian Yin, Tie-Yan Liu

**Affiliations:** 1 School of Data and Computer Science, Sun Yat-Sen University, Guangzhou, China; 2 Guangdong Key Laboratory of Big Data Analysis and Processing, Guangzhou, China; 3 Microsoft Research Asia, Beijing, China

**Keywords:** Fragment library, Structural information, Protein property prediction, Protein folding

## Abstract

**Background:**

Fragment libraries play a key role in fragment-assembly based protein structure prediction, where protein fragments are assembled to form a complete three-dimensional structure. Rich and accurate structural information embedded in fragment libraries has not been systematically extracted and used beyond fragment assembly.

**Methods:**

To better leverage the valuable structural information for protein structure prediction, we extracted seven types of structural information from fragment libraries. We broadened the usage of such structural information by transforming fragment libraries into protein-specific potentials for gradient-descent based protein folding and encoding fragment libraries as structural features for protein property prediction.

**Results:**

Fragment libraires improved the accuracy of protein folding and outperformed state-of-the-art algorithms with respect to predicted properties, such as torsion angles and inter-residue distances.

**Conclusion:**

Our work implies that the rich structural information extracted from fragment libraries can complement sequence-derived features to help protein structure prediction.

**Supplementary Information:**

The online version contains supplementary material available at 10.1186/s12859-021-04258-6.

## Background

Protein structure prediction is one of the most challenging and active research fields in computational biology [1–3]. Over decades of research, fragment assembly proved to be one of the most successful ab initio approach [2, 4] although the latest end-to-end solution AlphaFold2 brought disruptive progress [5]. Fragment assembly has been widely used in many popular systems, such as Rosetta [6] and Quark [7]. The high-quality fragment libraries are one of the determining factors to the success of fragment assembly [8, 9]. Many fragment library construction algorithms such as NNMake [10], LRFragLib [8], Flib-Coevo [11], and DeepFragLib [12] have been proposed to recruit as many near-native fragments as possible for each position of the target protein. Fragment libraries contain rich structural information, including 1D structural properties such as secondary structure and torsion angles, and 2D structural properties such as distances and orientations between pairs of heavy atoms. Although fragment libraries are extensively utilized in fragment assembly, the rich structural information has not yet been systematically analyzed and leveraged by other protein structure prediction approaches.

Exemplified by AlphaFold [13], trRosetta [14], and GDFold [15], recent research heavily used gradient descent to fold protein structures by optimizing potentials derived from predicted protein properties, such as C_β_ − C_β_ pairwise distances and torsion angles. These approaches generally consist of two stages: in the protein property prediction stage, many types of structural properties are predicted for a protein sequence; in the gradient-descent based protein folding stage, structures are generated by minimizing the energy potential derived from protein properties. Given that the energy potentials are mainly derived from the predicted protein properties, the accuracy of the predicted protein properties, to a large extent, determines the quality of final predicted structures.

In recent research and industrial pipelines of protein property prediction, such as the prediction of secondary structures [16, 17], torsion angles [16] and inter-residue distances [18], the most widely used features, including sequence profiles and multiple sequence alignments, are derived from protein sequences. Such features, when coupled with carefully designed algorithms, lead to good predictions empirically. However, those features only leverage sequential information, and incorporating new features from known protein structures could serve as a complement and thus benefit protein property predictions. In addition, some recent works such as [19, 20] adopted structural information in other bioinformatics fields and the considerable performance gains indicate the huge potential of protein structural information.

In this study, to leverage the structural information provided by fragment libraries, we first directly extracted multiple structural properties from fragment libraries and proposed novel fragment-level metrics for evaluation. We used DeepFragLib, the state-of-the-art fragment library construction approach when benchmarked on recent CASPs [21], to generate fragment libraries for subsequent studies. Then we broadened the usage of such structural information by employing fragment libraries both as potentials for gradient descent-based protein folding and as input features of a deep learning model for protein property prediction. For protein folding, protein properties directly extracted from fragment libraries were fitted with a set of weighted Gaussian Mixture Models (wGMM) and then incorporated as protein-specific potentials into a gradient-descent based folding system, SAMF [22]. For protein property prediction, we designed FA-DNN, a deep neural network that encodes fragment libraries into features using a fragment library encoder and predicts multiple protein properties. Different from state-of-the-art protein property predictors such as SPOT-1D and Spider3 that only use sequential information to predict 1D properties, i.e., torsion angles and backbone angles, FA-DNN further takes the structural information extracted from fragment libraries and predicts both 1D properties and 2D properties (e.g., inter-residue distances) with a higher accuracy. The evaluation on CASP13 FM (free modelling), CASP13 TBM (template-based modelling), the “hard” targets from Continuous Automated Model Evaluation (CAMEO) [22] and the latest CASP14 FM showed that the incorporation of fragment libraries improved the performance of both protein property prediction and protein folding.

We summarize our findings as follows. First, our comprehensive analysis of fragment libraries clearly showed their potential in facilitating protein structure-related tasks. Second, our attempt to leverage fragment libraries as potential functions in gradient descent-based protein modelling pipelines brought small improvements in TM-Score, but it helped moderately in quality assessment. Finally, our proposed FA-DNN that employs fragment libraries as inputs showed superior prediction performance for torsion angles, backbone angles and Cβ distances when compared to state-of-the-art protein property predictors on four independent test sets.

## Results

### Rich and accurate structural information in fragment libraries

**Fig. 1:**
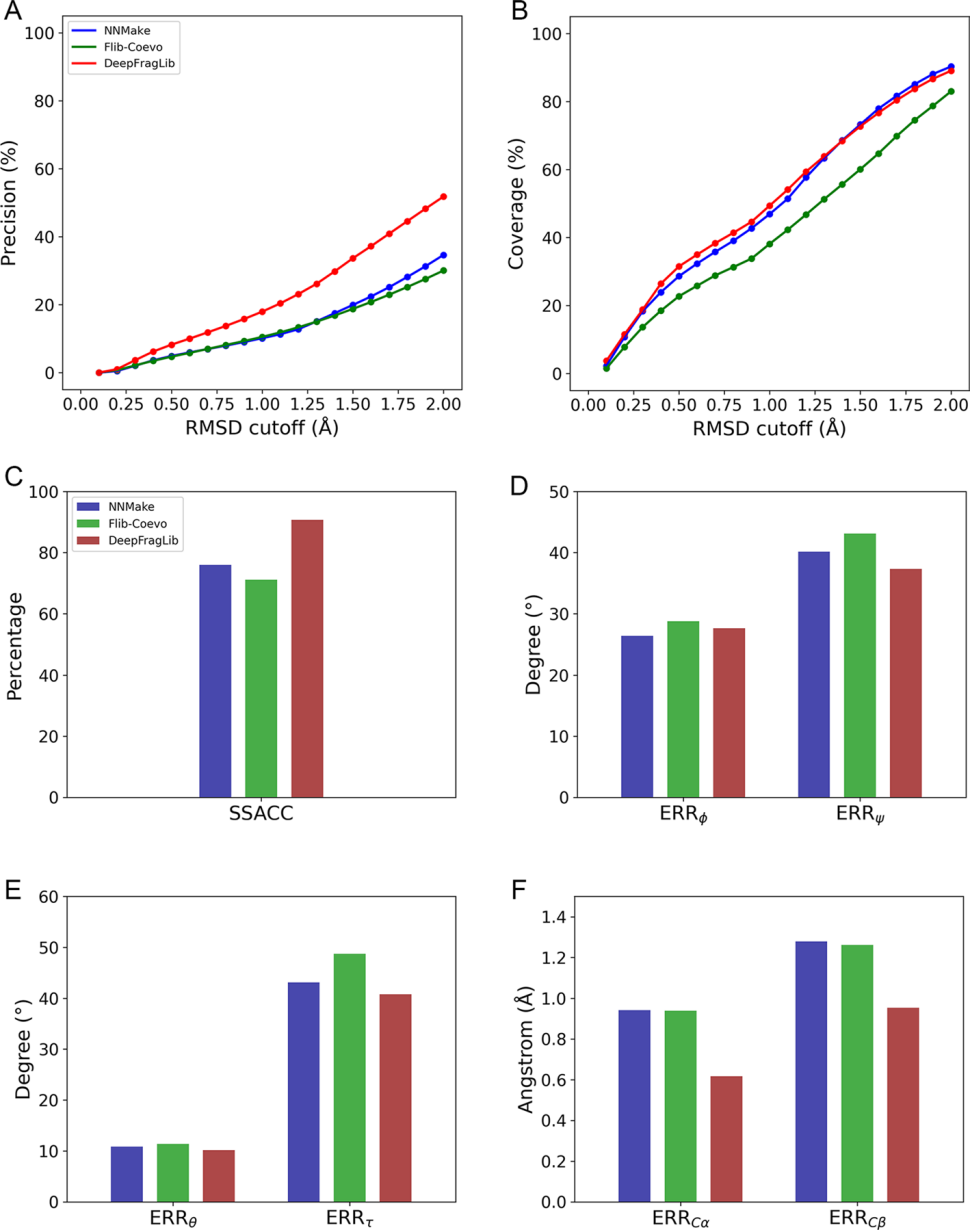
Quality analysis of fragment libraries on all targets of CASP13 FM, CASP13 TBM and CAMEO test sets. **a** and **b** Fragment libraries constructed by NNMake (blue), Flib-Coevo (green) and DeepFragLib (red) were evaluated using precision (**a**) and coverage (**b**) at a series of RMSD thresholds. **c**–**f** Fragment libraries were evaluated using fragment level metrics for seven structural properties, i.e., the accuracy of fragment secondary structure, the error of torsion angles, ϕ, ψ, the error of backbone angles, θ, τ and the error of C_α_ − C_α_ distances and C_β_ − C_β_ distances. See Additional file 1: Figures S1–S3 for performance on respective test sets

Initially designed to serve in fragment assembly-based simulations, fragment libraries are lists of short template structures (i.e., fragments) that are considered resemble to continuous regions of the structure of a target protein. Although a fragment library consists of thousands of fragments and thus contains rich structural information about the target protein, such information is lack of exploration and seldom used beyond fragment assembly. To obtain a quantitative understanding of structural information embedded in fragment libraries, we constructed fragment libraries for proteins in all three independent test datasets, CASP13 FM, CASP13 TBM and CAMEO [23] using three state-of-the-art algorithms, DeepFragLib [12], NNMake [10] and Flib-Coevo [11], respectively. The overall performance (Fig. 1a, b) on the three test sets were evaluated on two classical metrics, precision and coverage, at different RMSD cutoff values ranging from 0.1 to 2.0 Å. DeepFragLib outperformed NNMake and Flib-Coevo with a large margin on precision and achieved a coverage of about 90% at 2.0 Å cutoff, which indicates that DeepFragLib recruited much more near-native fragments for most of the positions of target proteins than other algorithms.

Although precision and coverage can be used to evaluate fragment libraries on an overall level, they generally fail to reflect the accuracy of detailed structural properties of a fragment library. To quantitively analyze the structural information of fragment libraries, we devised seven novel fragment-level metrics for corresponding structural properties, namely the accuracy of fragment secondary structure, the error of two torsion angles, ϕ and ψ, the error of two backbone angles θ and τ [24] and the error of C_α_ − C_α_ and C_β_ − C_β_ pairwise distances (see Methods for more details). These fragment-level metrics were defined as the expectations of errors (or accuracy for fragment secondary structure) of all positions of a target protein, where the expectation for each position was defined as errors (or accuracies) of structural properties of all fragments at this position. Therefore, different from previous metrics for protein property evaluation (e.g., the accuracy of secondary structure or the accuracy of torsion angles), which were directly defined on a single residue and evaluated for the target protein, our proposed metrics are all defined on the fragment level and thus can better evaluate fragment libraries constructed by different algorithms. As shown in Fig. 1c–f and Additional file 1: Figure S1–S3, seven kinds of structural properties were extracted from fragment libraries and analyzed using our proposed metrics. All fragment libraries achieved high accuracy on fragment secondary structure and low error values on other properties, with DeepFragLib outperforming the other two algorithms except for ERR_ϕ_. These results demonstrate that fragment libraries provide rich and high-quality structural information that is lack of evaluation before. To get more accurate structural information for protein structure prediction, we further assigned a confidence score for each fragment in DeepFragLib according to the predicted RMSD value using a softmax function with T = 0.1 and constructed weighted fragment libraries (see Methods for more details). As shown in Additional file 1: Table S1, all seven kinds of structural information derived from weighted fragment libraries are more accurate than those from vanilla fragment libraries built by DeepFragLib, which indicates the predicted RMSD value can work as a confidence score for extracting more accurate structural information from fragment libraries. Furthermore, we run AbInitioRelax from Rosetta v3.10 [25] to implement a fragment-assembly based protein folding pipeline with fragment libraries constructed by DeepFragLib and NNMake (the default fragment library construction algorithm in Rosetta), respectively (see Supplementary Material for more details). Results (Additional file 1: Table S2) show that DeepFragLib generally has better performance in fragment assembly. Considering that DeepFragLib outperformed the others in terms of both the accuracy of structural information and the applicability for protein structure prediction, we chose DeepFragLib to conduct the subsequent experiments in the study.

### Fragment libraries as potentials for gradient-descent based protein folding

**Fig. 2:**
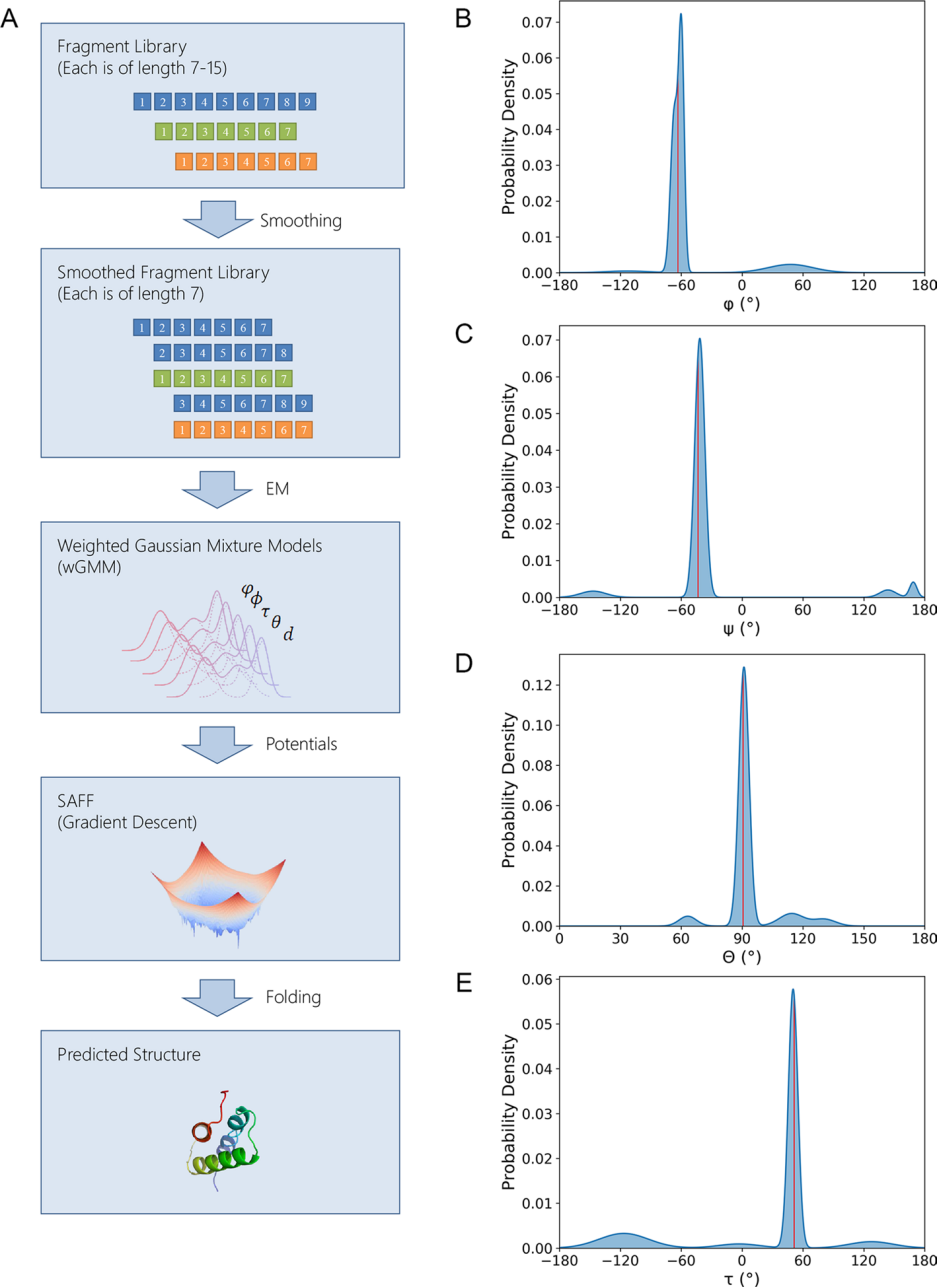
The overall pipeline of building wGMM models and protein-specific potentials from fragment libraries. **a** The overall pipeline that transforms fragment libraries into potentials for gradient descent-based protein folding. Fragments of variable lengths are first cut into a series of 7-residue fragments by smoothing. The color of a fragment denotes its “source” fragment (i.e., three fragments with 7 residues colored in blue in the second subfigure are all cut from the 9-residue fragment colored in blue in the first subfigure). Protein properties are extracted from the smoothed fragment library and fitted by weighted Gaussian mixture models (wGMM models). wGMM models are converted into potentials and utilized in SAMF [22] for protein folding. **b**–**e** Visualization of the wGMM models for ϕ (**b**), ψ (**c**), θ (**d**), τ (**e**) of the first residue of 67th position of T0969-D1. The red lines mark the corresponding properties of the native protein structure

Gradient-descent based protein folding is an approach that predicts protein 3D structures by directly minimizing protein property constraints. The quality of protein property constraints usually determines the accuracy of predicted structures. Considering that most constraints including inter-residue pairwise distances and torsion angles [13, 14] are derived from sequential information, we propose to complement these constraints with the structural information extracted from fragment libraries. Therefore, we devised an approach that transformed structural information of fragment libraries into protein-specific potentials, and folded protein structures with inter-residue distance potentials predicted by trRosetta. As shown in Fig. 2a, the approach starts with a “smoothing” operation that normalizes fragments of variable lengths to a series of sub-fragments with a fixed length of 7 residues by using a sliding window. Then, different kinds of protein properties in the smoothed fragment library are extracted and modeled by weighted Gaussian mixture models (termed as wGMM). Finally, these wGMM models are converted to protein-specific potentials by a negative log likelihood function and incorporated into the distance potential for protein structure prediction (see Methods for more details).

In this approach, six kinds of structural properties were extracted from fragment libraries, including two torsion angles (ϕ and ψ), two backbone angles (θ and τ) and two pairwise distances within a fragment (C_α_ − C_α_ and C_β_ − C_β_). A wGMM model was built for each property and each position of the target protein, respectively. To choose the number of components in each wGMM model, we fitted wGMMs on the torsion angle ϕ using a series of numbers of components ranging from 2 to 30 and used the averaged BIC (Bayesian Information Criterion) score as the criterion to evaluate on CASP12FM dataset. As shown in Additional file 1: Figure S4, wGMM models with four components had the lowest BIC score and thus four components were chosen for wGMM modeling. Figure 2b–e illustrates an example (the first residue of the 67th position of T0969-D1) of the distributions in wGMM models of the torsion and backbone angles. Each parameterized distribution colored in blue forms a peak, and the ground truth of the corresponding property colored in red is very close to the peak in each model, demonstrating that it is reasonable to employ wGMM models to fit the structural information extracted from fragment libraries.

To evaluate the usefulness of structural information for protein folding, we benchmarked this approach on all three independent test sets. Taking distance constraints generated by trRosetta as a base potential, 50 decoys were generated for each target protein with or without the potentials derived from the fragment wGMM models respectively, and all decoys were ranked according to their energies assigned by the sum of all potentials. The top1 decoys with lowest energies and the best decoys with highest TM-Score values were both picked up, and two metrics including the averaged TM-Score of all targets and the number of targets that have correct topologies, i.e. with TM-Score greater than 0.5 [26, 27], were used for evaluation. As shown in Table 1, when evaluated on all independent test sets, predicted structures with wGMM models outperformed those without wGMM models in all four metrics and especially an improvement of 8.84% was achieved on average TM-Score of top1 decoys. This result indicates that structural information of fragment libraries modelled by wGMMs improved the accuracy of protein structure prediction. Since protein targets considerably vary in terms of topologies, predicted protein structures within a test set exhibit a wide range of TM-Score values, which results in a moderate standard deviation. To further examine the significance of improvement when adopting fragment derived potentials, we then performed one-sided paired student T-tests on all protein targets in the three test sets (the test set denoted as “Overall” in Table 1). The *p* values for the best models and the top1 models are 0.01315 and 0.00098, respectively, which indicates the improvement on predicted protein structures is significant. Furthermore, it is worth noting that using potentials from fragment libraries narrowed the gap of TM-Score values between the best decoys and top1 decoys from 0.1189 to 0.0827, which indicates that the potentials derived from fragment libraries not only have their value on predicting more accurate protein structures, but also help to select more native structures among predicted decoys.

**Table 1:** Overall performance of protein folding with potentials derived from fragment libraries

Test set	Potentials	Avg. TM of Best (± s.d.)	Avg. TM of Top1 (± s.d.)	# Best with TM > 0.5	# Top1 with TM > 0.5
CASP13 FM	trRosetta Cβ dist	0.5523 (± 0.1154)	0.4572 (± 0.1547)	22	**15**
	trRosetta Cβ dist + FragLib wGMM	**0.5689 (± 0.1082)**	**0.4660 (± 0.1647)**	23	**15**
CASP13 TBM	trRosetta Cβ dist	**0.6619 (± 0.1069)**	0.4806 (± 0.2119)	**53**	26
	trRosetta Cβ dist + FragLib wGMM	0.6610 (± 0.1098)	**0.5806 (± 0.1800)**	52	**43**
CAMEO	trRosetta Cβ dist	0.5932 (± 0.1326)	0.4953 (± 0.1779)	101	70
	trRosetta Cβ dist + FragLib wGMM	**0.6011 (± 0.1322)**	**0.5212 (± 0.1765)**	**102**	**78**
Overall	trRosetta Cb dist	0.6051 (± 0.1279)	0.4862 (± 0.1839)	176	111
	trRosetta Cβ dist + FragLib wGMM	**0.6119 (± 0.1278)**	**0.5292 (± 0.1788)**	**177**	**135**

Potentials derived from fragment libraries were utilized to predict protein structures with trRosetta distance potential as a baseline. For each target, the top1 decoy with lowest energy and the best decoy with the highest TM-Score were picked up for evaluation. The mean and standard deviation of TM-Scores of all targets and the number of selected decoys with TM-Score > 0.5 were evaluated. The better performance in each category is highlighted in bold

### Fragment libraries as features for protein property prediction

Besides directly utilizing fragment libraries to facilitate protein structure prediction, the structural information extracted from fragment libraries can also serve as features for protein property prediction. For this purpose, we designed FA-DNN (Fragment-Assisted Deep Neural Network), a deep neural network consisting of a fragment library encoder module and a structural property predictor module (Additional file 1: Figure S5). The fragment library encoder takes a fragment library as input and encodes its structural information via a deep neural network. The protein property predictor adopts the hidden representations output by the fragment library encoder as well as sequence-derived features as input and predicts multiple protein properties, namely the backbone torsion angles (ϕ, ψ, θ and τ), and C_β_ − C_β_ pairwise distances (see Methods for more details). We first built a dataset called HR5916 by culling 5916 high-quality chains from PDB using PISCES [28] and randomly divided them into a training set and a validation set, which contained 90% and 10% of the chains, respectively. All hyperparameters were optimized on HR5916 validation set. For fair comparison, we also designed a baseline model by only removing the fragment encoder. The baseline model was trained with the same hyperparameters as control. The performance of FA-DNN and the baseline model was evaluated on three independent test sets, namely CASP13 FM, CASP13 TBM and CAMEO. A parameter-less model that simply averaged the property values from fragment libraries was also evaluated. Notably, considering that this model provides the prediction of inter-residue distances only for intra-fragment residue pairs, it cannot be compared with the other two models on C_β_ − C_β_ distance prediction.

**Fig. 3:**
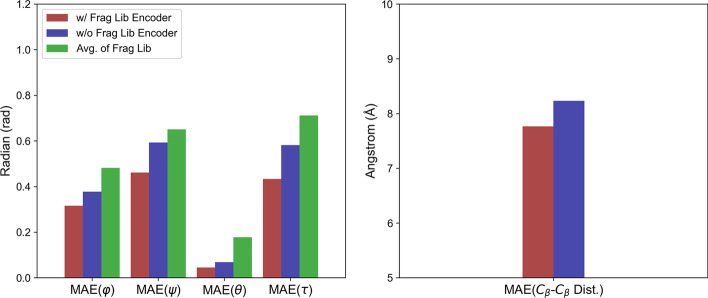
Performance analysis of FA-DNN for protein property prediction on three independent test sets. The FA-DNN consists of a fragment library encoder and a protein property predictor for protein property prediction (red bars). As control, a model without the fragment library encoder was trained with the same hyperparameters (blue bars). The performance of the two neural networks were evaluated on all targets of the three test sets. Additionally, performance of simply extraction from fragment libraries was also evaluated (green bars). The mean absolute error (MAE) of torsion angles (ϕ, ψ, θ and τ) and the MAE of C_β_ − C_β_ pairwise distances are shown in the left and right subfigures, respectively

We evaluated the accuracy of the real-valued predictions of protein properties by mean absolute error (MAE). As shown in Fig. 3, when evaluated on all targets of CASP13 FM, CASP13 TBM and CAMEO test sets (respective evaluation shown in Additional file 1: Figure S6–S8), FA-DNN outperformed the baseline model with a large margin in terms of the accuracy of all four torsion angles, while simply averaging protein 1D properties from fragment libraries achieved the poorest performance. For prediction of pairwise C_β_ − C_β_ distances, incorporating features of fragment libraries led to a moderate improvement in accuracy.

We further compared the performance of FA-DNN and state-of-the-art algorithms for protein property prediction. As shown in Table 2, FA-DNN outperformed Spider3 with a large margin in all four 1D properties. It is worth noting that although FA-DNN is a single deep learning model, it achieved better performance than SPOT-1D that is an ensemble of multiple models. Employing an ensemble of multiple FA-DNNs may further improve the performance. For 2D predictions, FA-DNN outperformed two state-of-the-art algorithms RaptorX and trRosetta in terms of the MAE (mean average error of real value) of C_β_ − C_β_ distances with a large margin.

**Table 2:** Comparison of predicted protein properties between FA-DNN and state-of-the-art algorithms on three independent test sets

Category	SPOT-1D	Spider 3	RaptorX	trRosetta	FA-DNN
MAE (ϕ)	0.312	0.337	–	–	**0.303**
MAE (ψ)	0.430	0.495	–	–	**0.429**
MAE (θ)	**0.041**	0.062	–	–	**0.041**
MAE (τ)	0.428	0.479	–	–	**0.418**
MAE (C_β_ − C_β_ Dist.)	–	–	8.636	8.304	**7.766**

The MAE of real values of protein properties were evaluated. The best performance of each category is shown in bold

**Fig. 4:**
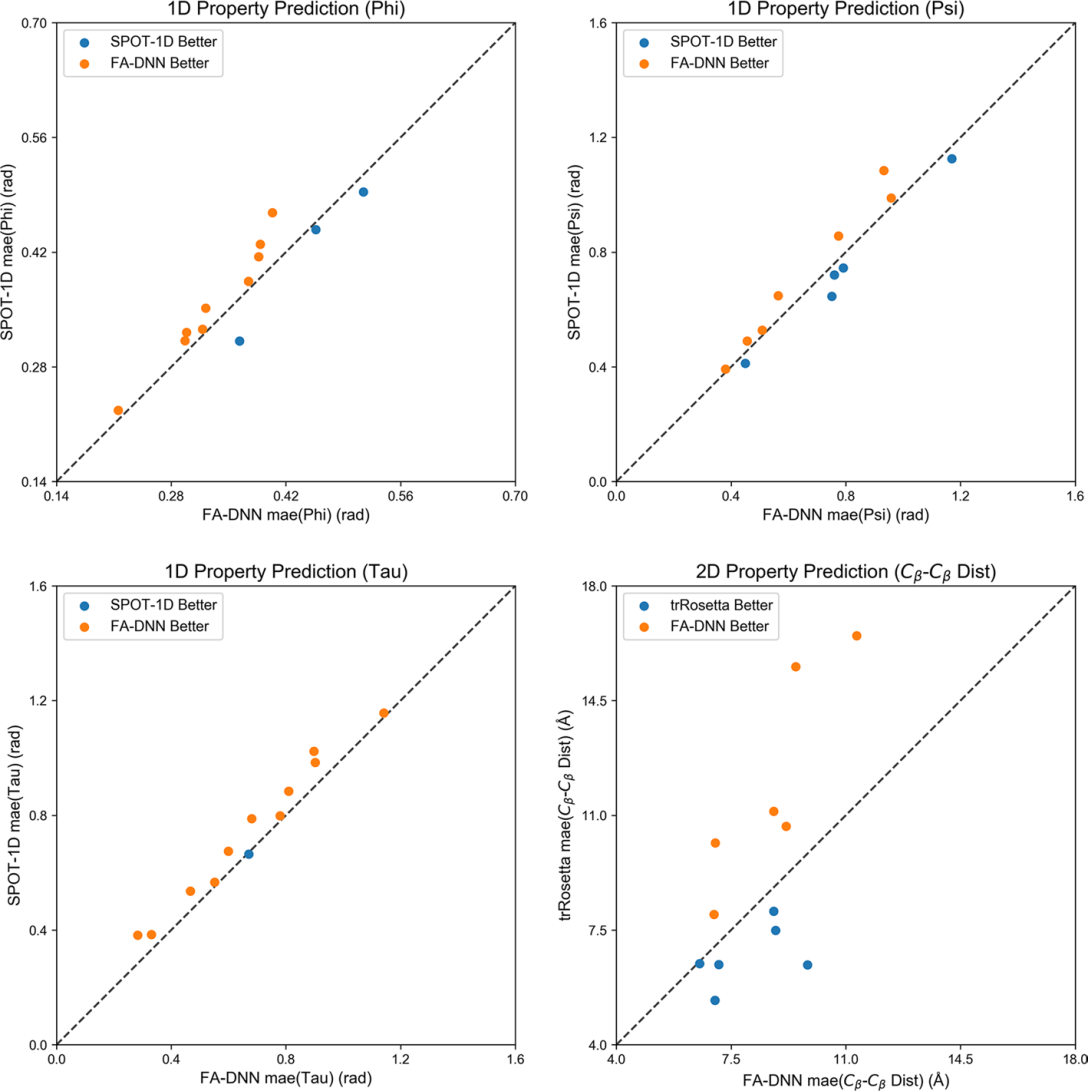
Point-to-point comparison between FA-DNN and state-of-the-art protein property prediction algorithms on CASP14 FM targets

We further made a point-to-point comparison between our FA-DNN with SPOT-1D and trRosetta on the latest CASP14 FM test set. As is shown in Fig. 4, FA-DNN had a small improvement over SPOT-1D on most CASP14 FM targets, and outperformed trRosetta in general, with large improvements on two targets, T1037 and T1042. Careful examination of the prediction pipelines shows that each of the two targets has very limited number of multiple sequence alignments (MSA) and trRosetta only uses MSA as input, leading to its degenerate performance on these two targets. As a comparison, using structural information from fragment library in FA-DNN may compensate the limited sequence information to some extent and thus leads to better predictions.

## Discussion and conclusion

This work, to our knowledge, is the first attempt to exploit structural information from high-quality fragment libraries for both protein property prediction and gradient-descent based protein folding. By explicitly extracting structural information from fragment libraries and designing corresponding potentials with wGMM models, we demonstrate that leveraging fragment libraries leads to performance gains for protein structure prediction. Therefore, it might be of interest to find out what leads to the improvements. One obvious observation is that fragment libraries provide rich structural information which works as constraints beyond those predicted from sequential information. A fragment library consists of many fragments for each position of the target protein, where each fragment is a local 3D structure, containing almost all kinds of structural properties like a protein can have. Different from constraints predicted from sequence-based information, such as pairwise inter-residue distances, which mainly provide global constraints among residues, fragments capture short-ranged and local structural information. Therefore, constraints extracted from fragment libraries and those predicted from sequence-derived information, are complementary to each other. Nonetheless, we found that such local structural information can only bring limited improvement for protein folding and the gap to the best approach in the CASP competitions is still large. In this study, due to the limited number of fragments, the wGMM models were built for each kind of protein property and for each position of a target protein respectively. Taking into consideration the interactions among different protein properties and different positions, a potentially better way is to employ multivariate wGMMs to model such properties and thus produce more accurate potentials for the subsequent gradient-based protein folding.

With its information implicitly extracted by a neural network, fragment library also makes a difference in protein property prediction. Considering the quality of fragment libraries for different proteins varies from one to another, we further estimated the relationship between the quality of fragment libraries and the performance gains of protein property prediction. As shown in Additional file 1: Figure S9, the precision of fragment library is highly correlated to the averaged value of MAE of three torsion angles (ρ =  − 0.801 without two outliers T0955-D1 and T1008-D1), indicating a nearly linear negative correlation between these two metrics. Therefore, recruiting more high-quality fragments for target proteins is an essential way to improve the accuracy of predicted protein properties.

Although both the fragment derived potentials and FA-DNN with fragment libraries as inputs showed effectiveness on their own, how to fully utilize the advantages from the two aspects and avoid redundancies still requires further study. An end-to-end model that integrates the two approaches in a single deep neural network and simultaneously optimizes predicted properties and 3D structures, can be expected in the future.

## Methods

### Datasets

To evaluate the performance of fragment libraries, we adopted three independent test sets, including 31 targets in CASP13FM (Additional file 1: Table S3), 56 targets in CASP13TBM (Additional file 1: Table S4) and 137 targets in CAMEO (Additional file 1: Table S5). Specifically, CASP13FM and CASP13TBM consist of all free modeling (FM) domain targets and all template-based modeling (TBM) domain targets extracted from the official website of CASP13 competition [21], respectively. To test the robustness of our approaches for different proteins and make evaluation on more targets, we culled all targets in the “hard” category from CAMEO official website [23] released within the year of 2019 (from 2019.01.04 to 2019.12.28). For dataset construction, targets with discontinuous chains, targets whose lengths are less than 40 residues and targets which Flib-Coevo failed to construct fragment libraries were discarded, leading to a slightly smaller number of targets than that in the corresponding official websites.

CASP13FM test set was first used to evaluate the performance of protein structure prediction by Rosetta while all three independent test sets were then employed to assess the effectiveness and contribution of fragment libraries in the gradient-descent based protein folding software, SAMF [22]. In addition, for optimization of potentials derived from fragment libraries in SAMF, we built a dataset from CASP12FM proteins (Additional file 1: Table S6) which included 34 FM domain targets with continuous chains extracted from CASP12 competition [29].

To train the deep neural network including a fragment library encoder and a protein property predictor for protein property prediction, we culled 5916 high resolution protein chains from PISCES (termed as “HR5916”) [28] with chain length < 400 residues, resolution < 1.5 Å, pairwise sequence identity < 30% and PDB release date before CASP13 competition (2018.05.01). We randomly chose 5325 chains to build the training set and the remaining 591 chains formed the validation set. CASP13FM, CASP13TBM and CAMEO were also utilized as independent test sets for performance evaluation. Furthermore, twelve CASP14 FM targets (Additional file 1: Table S7) were also used in the performance comparison between FA-DNN and state-of-the-art algorithms. Notably, considering that all samples in CASP12FM and HR5916 are before CASP13 competition, there is no overlap between the training samples and the independent test sets, which ensures a fair evaluation. As a sanity test, for each target in the test sets, we performed an MSA search against Lib956, the template structure library from which DeepFragLib recruits fragments, with jackhmmer [30] 3.3.1 and an E-value of 1e−3. The average MSA depths (the number of detected homologous proteins), as shown in Additional file 1: Table S8, are smaller than 1.0 for all test sets, which indicates for most targets, no homologous structure can be found in Lib956 and thus confirms that the test sets have no overlap with the fragment libraries.

### Fragment library construction and comprehensive analysis

We built fragment libraries for all targets in CASP13FM, CASP13TBM and CAMEO test sets by DeepFragLib v1.0 [12]. 50–200 fragments with known structures were recruited for each position of the target protein. During the construction process, all databases queried by DeepFragLib were strictly restricted to the archived versions with timestamps before CASP13 competition. For performance evaluation, we also built fragment libraries for these test sets by NNMake [10] and Flib-Coevo [11] with their default parameters, respectively. Duplicate fragments at the same position generated by Flib-Coevo were excluded for fair comparison. All fragment libraries constructed by different algorithms were converted to the uniform NNMake’s format for further analysis.

We first assessed the performance of fragment libraries constructed by different algorithms with two classical evaluation metrics, namely precision and coverage. Precision is the proportion of good fragments in the whole fragment library and coverage is the proportion of positions in each protein which are spanned by at least one good fragment. A good fragment is defined as a fragment with an RMSD smaller than a given threshold. Furthermore, in order to make a comprehensive evaluation on the structural properties of fragment libraries, we took seven protein structure properties into consideration, namely the 1D properties including the secondary structures, the torsion angles ϕ, ψ, the backbone angles θ, i.e., the planar angle between three successive Cα atoms (${\text{C}}_{{\upalpha }}^{{{{\rm i}} - 1}} - {\text{C}}_{{\upalpha }}^{{{\rm i}}} - {\text{C}}_{{\upalpha }}^{{{{{\rm i} + }}1}}$) and τ, i.e., the dihedral angle between four successive Cα atoms (${\text{C}}_{{\upalpha }}^{{{{\rm i}} - 1}} - {\text{C}}_{{\upalpha }}^{{{\rm i}}} - {\text{C}}_{{\upalpha }}^{{{{{\rm i} + }}1}} - {\text{C}}_{{\upalpha }}^{{{{{\rm i} + 2}}}}$) and the 2D properties including the C_α_ − C_α_ and C_β_ − C_β_ pairwise distances. Though these properties were originally defined on the residue level, we proposed novel metrics describing the accuracy of these properties on the fragment level.

Similar to previous research, the secondary structures of fragments are divided into four classes: mainly helix (termed as H), mainly strand (termed as E), mainly coil (termed as C) and others (termed as O). A fragment is defined as H or E or C if more than half residues of the fragment are assigned with the corresponding secondary structures. Otherwise, the secondary structure of the fragment is defined as O. The accuracy of fragment secondary structure on the fragment level is defined as follows,

1 $$\begin{array}{*{20}c} {I_{{SS}} \left( {f_{i} } \right) = \left\{ {\begin{array}{*{20}l} {1,} \hfill & {\quad if\;SS\left( {f_{i} } \right) = SS\left( {f_{*} } \right)} \hfill \\ {0,~} \hfill & {\quad otherwise} \hfill \\ \end{array} } \right.} \\ \end{array}$$

2 $$\begin{array}{*{20}c} {ACCss\left( {FL} \right) = E_{{p_{i} }} \left[ {E_{{f_{i} }} \left[ {{\text{I}}_{{SS}} \left( {f_{i} } \right)} \right]} \right]} \\ \end{array}$$

where FL denotes the fragment library, E denotes the mathematical expectation, p_i_ denotes all fragments at position i, f_i_ denotes a fragment at position i, f_*_ denotes the corresponding fragment of the target protein and SS(f) denotes the fragment secondary structure of fragment f. Thus, the accuracy of fragment secondary structure of the whole fragment library ACC_SS_(FL) is defined as the expectation of the accuracy of each position, which is then defined as the expectation of the accuracy of all fragments at this position. Similar to the above definition, we further proposed the accuracy of angles (ϕ, ψ, θ and τ) as follows,

3 $$\begin{array}{*{20}c} {err_{{ang}} \left( {f_{i} ,f_{*} } \right) = \frac{1}{N}\mathop \sum \limits_{{j = 1}}^{N} \min \left\{ {\left| {ang_{i}^{j} - ang_{*}^{j} } \right|,360^\circ - \left| {ang_{i}^{j} - ang_{*}^{j} } \right|} \right\}} \\ \end{array}$$

4 $$\begin{array}{*{20}c} {ERR_{{ang}} \left( {FL} \right) = E_{{p_{i} }} \left[ {E_{{f_{i} }} \left[ {err_{{ang}} \left( {f_{i} ,f_{*} } \right)} \right]} \right]} \\ \end{array}$$

where |x| denotes the absolute value of x, ${\text{ang}}_{{{\rm i}}}^{{{\rm j}}}$ denotes the angle value of residue j of the fragment i, ${\text{ang}}_{*}^{\rm j}$ denotes the angle value of the corresponding residue in the target protein and err_ang_(f_i_, f_*_) denotes the mean absolute error of the angle of the fragment i. The angle error of a fragment library is defined as the expectation of those of all positions, where the angle error of a position is then defined as the expectation of those of all fragments at this position. Finally, we proposed the accuracy of two 2D properties (C_α_ − C_α_ and C_β_ − C_β_ pairwise distances) as follows,

5 $$\begin{array}{*{20}c} {ERR_{{dist}} \left( {FL} \right) = E_{{p_{i} }} \left[ {E_{{f_{i} }} \left[ {err_{{dist}} \left( {f_{i} ,f_{*} } \right)} \right]} \right]} \\ \end{array}$$

where err_dist_(f_i_, f_*_) denotes the mean absolute error (MAE) between pairwise C_α_ − C_α_ or C_β_ − C_β_ distances within a fragment f_i_ compared with the native structure f_*_. With these novel metrics, we made a comprehensive evaluation of fragment libraries constructed by NNMake, Flib-Coevo and DeepFragLib on three independent test sets.

### Fragment library for gradient-descent based protein folding

As illustrated above, we extracted and analyzed structural information embedded in fragment libraries. To further exploit the rich information from fragment libraries for gradient-descent based protein folding, we built a series of models to make an explicit representation of fragment libraries and then utilized these models to design protein-specific potentials in a differentiable way. As shown in Fig. 2a, six 1D and 2D properties of each fragment including the angles of ϕ, ψ, θ, τ and the pairwise distances between C_α_ − C_α_ and C_β_ − C_β_ atoms were extracted from the fragment library. Considering that fragments have variable lengths, we designed a “smoothing” operation which cuts fragments into a series of 7-residue long fragments by a sliding window. This operation results in all fragments having the same length of 7 residues. We employed Gaussian mixture models to delineate the distribution of these properties for each position. Considering that each fragment recruited by DeepFragLib has a predicted RMSD, we regarded this value as a confidence score for the fragment and assigned a weight according to all fragments at the same position as follows,

6 $$\begin{array}{c} {w_{{f_{i} }} = \frac{{e^{{\left( {5.0 - predRMSD_{i} } \right)/T}} }}{{\mathop \sum \nolimits_{{f_{j} }}^{F} e^{{\left( {5.0 - predRMSD_{j} } \right)/T}} }}} \\ \end{array}$$

where F denotes the set of fragments at the same position, f_i_ denotes a fragment in F, predRMSD_i_ denotes the predicted RMSD value of fragment f_i_ and T is the temperature (0.1 is used throughout the experiments).

7 $$\begin{array}{c} {p\left( {y;\mu ;{\upsigma }} \right) = \frac{1}{{\sqrt {2\pi \left| \sigma \right|} }}\exp \left( { - \frac{{\left( {y - \mu } \right)^{2} }}{{2\sigma ^{2} }}} \right)} \\ \end{array}$$

Furthermore, the probability density function of a Gaussian distribution is shown in Eq. 7, where y is the value of a property weighted by the ${\text{w}}_{{{\text{f}}_{{\text{i}}} }}$ in Eq. 6, µ denotes the weighted mean and σ^2^ denotes the weighted variance. We then built weighted Gaussian mixture (wGMM) models of each property for each residue with four components (Fig. 2). Consequently, for each position, seven wGMM models for each of four 1D properties and 21 wGMM models for each of two 2D properties were constructed, resulting in 70 wGMM models in total.

wGMM models were then converted to potentials using a negative log likelihood function. Notably, fragment-derived potentials are customized for each protein owing to protein-specific wGMM models. For example, the protein-specific loss function of ϕ angles (1D property) and inter-residue C_β_ − C_β_ distances (2D property) are defined as follows,

8 $$\begin{array}{c} {L_{\varphi } \left( x \right) = - \mathop \sum \limits_{i} log\mathop \sum \limits_{{t = 1}}^{K} w_{{i,t}}^{\varphi } p\left( {\varphi _{i} ;\mu _{{i,t}}^{\varphi } ;{\upsigma }_{{i,t}}^{\varphi } } \right)} \\ \end{array}$$

9 $$\begin{array}{c} {L_{{C_{\beta } }} \left( x \right) = - \mathop \sum \limits_{{j_{1} < j_{2} }} log\mathop \sum \limits_{{t = 1}}^{K} w_{{i,j_{1} ,j_{2} ,t}}^{{C_{\beta } }} p\left( {d_{{j_{1} ,j_{2} }}^{{C_{\beta } }} ;\mu _{{i,j_{1} ,j_{2} ,t}}^{{C_{\beta } }} ;{\upsigma }_{{i,j_{1} ,j_{2} ,t}}^{{C_{\beta } }} } \right)} \\ \end{array}$$

where Eq. 8 is the potential for ϕ, Eq. 9 is the potential for C_β_ − C_β_ distances, x denotes a predicted protein structure, K is the number of components in the wGMM model, w, µ and σ are the fitted parameters of each component of the wGMM model, ϕ_i_ is the ϕ angle at the i-th residue in x and ${\text{d}}_{\rm j1,j2}^{{\rm C}_{{\upbeta }}}$ is the distance between the C_β_ atom of the j_1_ residue and the C_β_ atom of the j_2_ residue in x. Potentials for other properties are defined in a similar way which led to a total of six potential functions (one for each property).

SAMF is a gradient descent-based protein folding framework that folds protein structures in a self-adaptive way [22]. To evaluate the performance of fragment library for gradient-descent based protein folding, we employed the most basic version of SAMF as the baseline, which only relies on C_β_ − C_β_ pairwise distances predicted by trRosetta [14] as protein-specific constraints, the fundamental geometry potentials to avoid stereo clashes as well as a naïve quality analysis module that sums all potentials. We implemented the potentials of the fragment library in SAMF with the combined potential function L_FL_(x) as follows,

10 $$\begin{array}{*{20}c} {L_{{FL}} \left( x \right) = w_{\varphi } L_{\varphi } \left( x \right) + w_{\psi } L_{\psi } \left( x \right) + w_{\theta } L_{\theta } \left( x \right) + w_{\tau } L_{\tau } \left( x \right) + w_{{C_{\alpha } }} L_{{C_{\alpha } }} \left( x \right) + w_{{C_{\beta } }} L_{{C_{\beta } }} \left( x \right)} \\ \end{array}$$

where L_FL_(x) is defined as the weighted sum of the six potentials and w denotes the weight of a potential for each property. The combined potential was calculated and then minimized to update the protein structure during each step of the gradient descent process. All weights in Eq. 10 were manually tuned on CASP12FM set by maximizing the mean TM-Score of predicted structures. The performance of protein structure prediction was then evaluated on CASP13FM, CASP13TBM and CAMEO test sets.

### FA-DNN: the fragment library encoder and the protein property predictor

To facilitate protein property prediction, we designed FA-DNN, a deep neural network featuring a fragment library encoder that implicitly extracts high-level representations of a fragment library and encodes it into an embedding vector. The fragment library encoder is connected with a protein property predictor which predicts multiple protein structural properties (Additional file 1: Figure S5A). For each position, we picked up 50 fragments with the lowest predicted RMSD values and extracted six kinds of features, namely the one-hot representation of residue secondary structure and the sine and cosine values of torsion angle ϕ, ψ, θ and τ. We padded all fragments of variable lengths to 15-residues long and the fragment library for a target protein was represented as an L × 50 × 15 × D tensor accordingly, where L denotes the length of the protein and D denotes the dimension of features. As shown in Additional file 1: Figure S5B, the fragment library encoder had a hierarchical architecture which contained three levels of encoding process. First, two 1D convolutional operations in each building block were implemented on the third dimension of the input tensor (the dimension of fragment length). A convolution kernel with the size of 3 and 64 filters were utilized in each convolutional operation and an ELU activation layer was adopted between two convolutional layers [31]. In order to fully learn the interactions between neighboring residues within a fragment, a total of 8 blocks were stacked with residual connections [32]. Considering that the index of the first residue of a fragment corresponds to the position of the target protein, the hidden representation of the first residue of each fragment was picked up, eliminating the dimension of the fragment length of tensors. Finally, an L × D’ output tensor was obtained by averaging the entries of all 50 fragments at the same position, where D’ is the number of filters of convolutional layers in the first step.

We then designed a protein property predictor which took the output of fragment library encoder as an input. In addition, the primary sequence of target protein, the position-specific frequency matrix (PSSM) of homologous proteins detected by DeepMSA [33] and the pairwise statistics derived from direct coupling analysis (DCA), were also fed into the predictor. The 1D features, namely all inputs from the fragment library encoder as well as the one-hot encoding of the target sequence and PSSM were transformed into two dimensions by tiling both horizontally and vertically, which were then concatenated with the pairwise statistics to form the total input of the predictor model. To examine the usefulness of the fragment library encoder, we also built a baseline predictor model as control which only takes the MSA-based feature as inputs.

Both the protein property predictor with inputs from the fragment library encoder and the baseline model share the same backbone architecture, i.e., a 2D residual neural network with 30 residual blocks and each residual block consisting of two convolutional layers with 64 filters, 3 × 3 kernel size and ELU activations. To prevent overfitting, a dropout rate of 0.15 [34] was used and two BatchNorm layers [35] were adopted after each convolutional layer (Additional file 1: Figure S5C). The output of the final residual block was symmetrized and then fed into two respective branches to predict different protein properties. The first branch began with a pooling operation to project the 2D feature map into an 1D vector. Following this operation, a fully connected layer was adopted to output 1D predicted properties of each residue similar to the raw feature from the fragment library, namely the four torsion angles φ, ψ, θ and τ. The other branch directly predicted the real value of C_β_ − C_β_ distances respectively by a full-connected layer. To favor the gradient for residue pairs with short distances, we employed a distance mapping function similar to [36] as follows,

11 $$\begin{array}{*{20}c} {d_{{i,j}}^{{\text{'}}} = tanh\left( {\frac{{d_{{i,j}} - 10}}{{2.4}}} \right)} \\ \end{array}$$

where d_i,j_ denotes the true distance between two atoms, ${\text{d}}_{{{\text{i,j}}}}^{\prime }$ denotes the mapped distance and tanh denotes the hyperbolic tangent function. The 2D outputs from the predictor were mapped back by the inverse function of Eq. 11 to get the corresponding real-valued distances. A weighted sum of the mean absolute errors (MAE) of all properties was used as the loss function. FA-DNN was trained on the HR5916 dataset with a learning rate of 1e−5. It took about one day to train the model on 8 Nvidia V100 GPUs. The baseline model was trained with the same hyper-parameters. The performance of all 1D and 2D predictions was evaluated on CASP13FM, CASP13TBM, CAMEO and CASP14 FM test sets.

## Supplementary Information

**Additional file 1**. Supplementary Method, Figure S1–S9 and Table S1–S8.

## Data Availability

All sequence data used in this study are publicly available. All data in CASP test sets can be downloaded at the official website of CASP: https://predictioncenter.org/index.cgi and data in CAMEO test set can be downloaded at the official website of CAMEO: https://www.cameo3d.org. The protein structures and fragment libraries of test sets generated by DeepFragLib are available at https://msracb.blob.core.windows.net/pub/data_for_complementing_with_fraglib.zip.
