# Supplementary material for: Complementing sequence-derived features with structural information extracted from fragment libraries for protein structure prediction
Source: BMC Bioinformatics. 2021 Jun 28;22:351. doi: 10.1186/s12859-021-04258-6 (PMC8240311; doi:10.1186/s12859-021-04258-6)
Supplement: Supplementary file 1 — Additional file 1. Supplementary Method, Figure S1–S9 and Table S1–S8. [file 12859_2021_4258_MOESM1_ESM.docx]

**Supplementary Material**

**Supplementary Method**

**Protein structure sampling by Rosetta**

We adopted AbInitioRelax from Rosetta v3.10 (1) to run ab initio protein structure prediction. AbInitioRelax is a fragment assembly-based protein structure prediction program which employs both short and long fragments generated by NNMake (2) to run a 4-stage Monte Carlo simulation, followed by a refinement procedure called Relax. Fragment libraries constructed by DeepFragLib (3) were converted to the fragment format of NNMake and then used in the program. To improve the accuracy of predicted structures, we increased the maximum steps in each simulation stage by a factor of ten. Two thousand predicted structures were generated for each target in CASP13FM test set, with fragment libraries derived from NNMake and DeepFragLib, respectively. We used TM-score (4) to evaluate the quality of predicted structures.


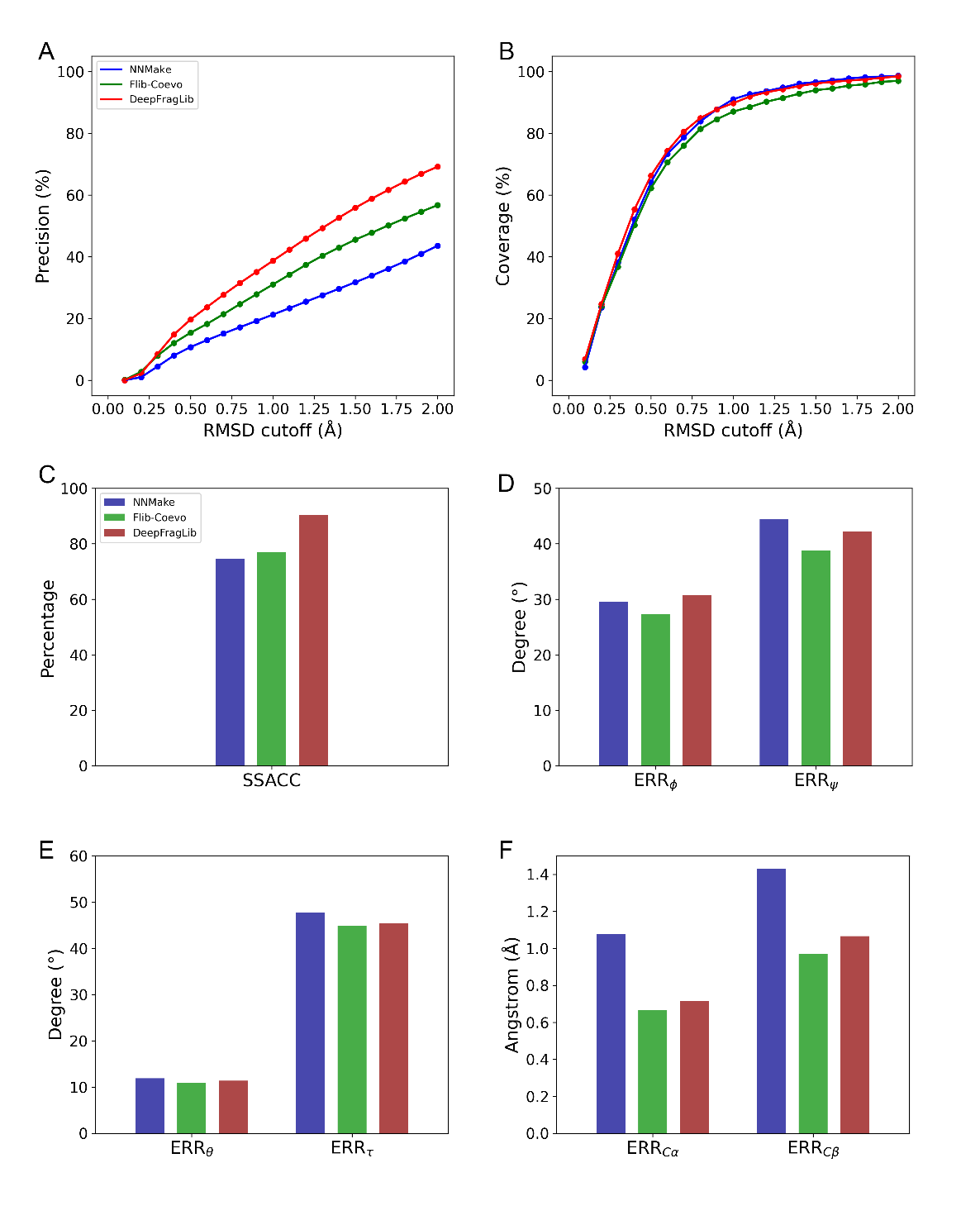


**Figure S1. Quality analysis of fragment libraries on CASP13 FM test set.**

A-B: Fragment libraries constructed by NNMake (blue), Flib-Coevo (green) and DeepFragLib (red) were evaluated using precision (A) and coverage (B) at a series of RMSD thresholds. C-F: Fragment libraries were evaluated using fragment level metrics for seven structural properties, i.e. the accuracy of fragment secondary structure, the error of torsion angles, ϕ, ψ, the error of backbone angles, θ, τ and the error of C_α_-C_α_ distances and C_β_-C_β_ distances.


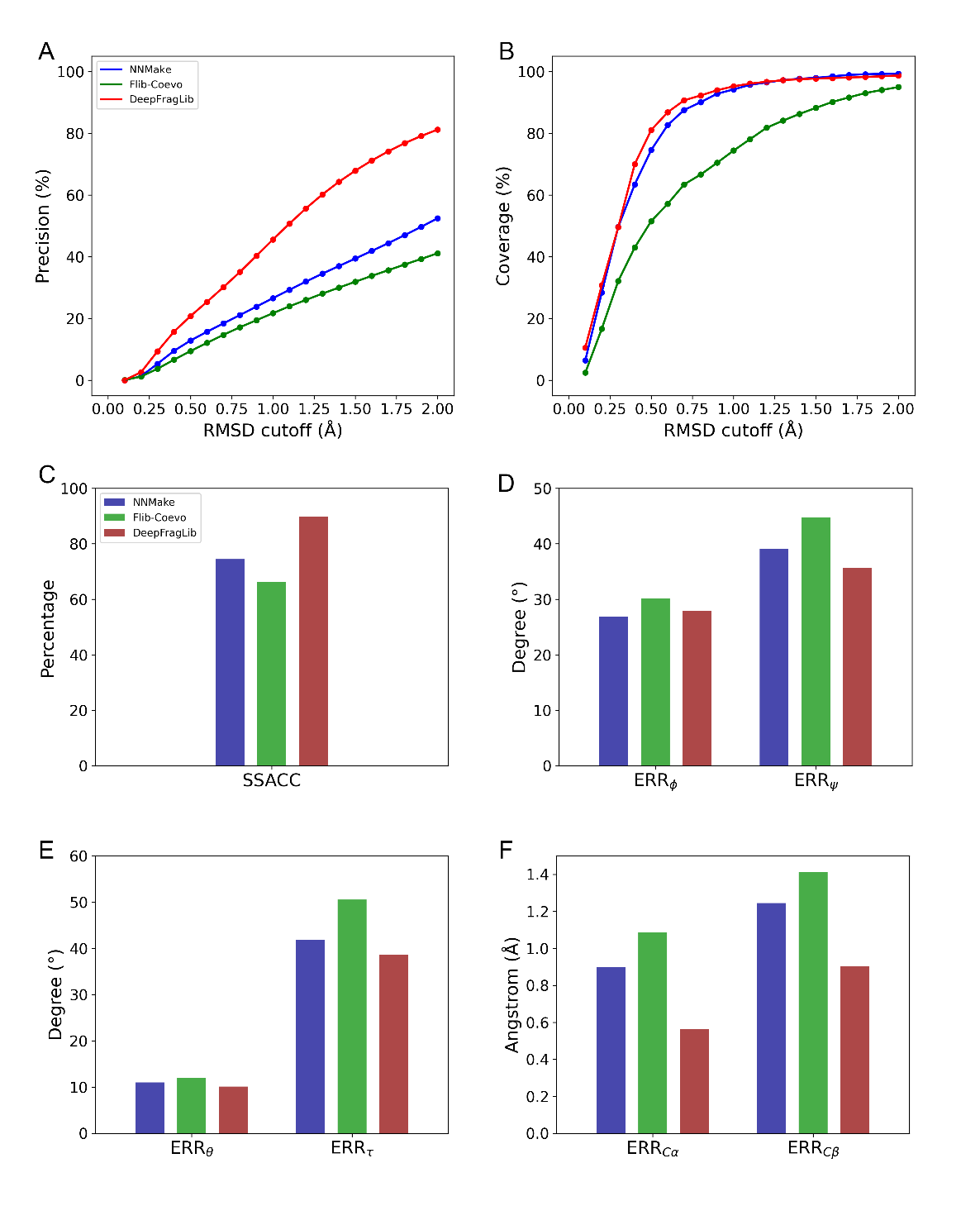


**Figure S2. Quality analysis of fragment libraries on CASP13 TBM test set.**

The categories and evaluation metrics on each subfigure are the same as those in Figure S1.


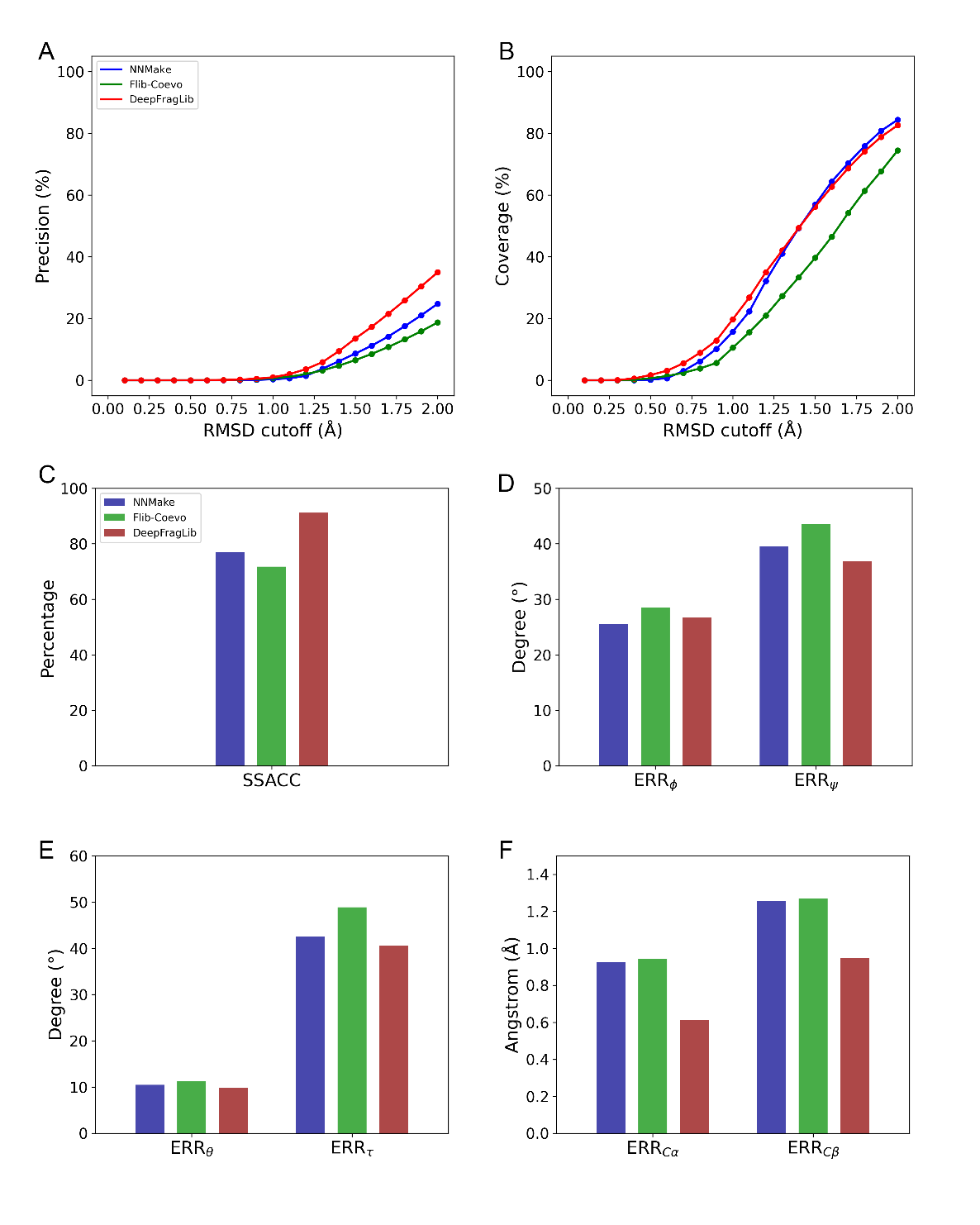


**Figure S3. Quality analysis of fragment libraries on CAMEO test set.**

The categories and evaluation metrics on each subfigure are the same as those in Figure S1.


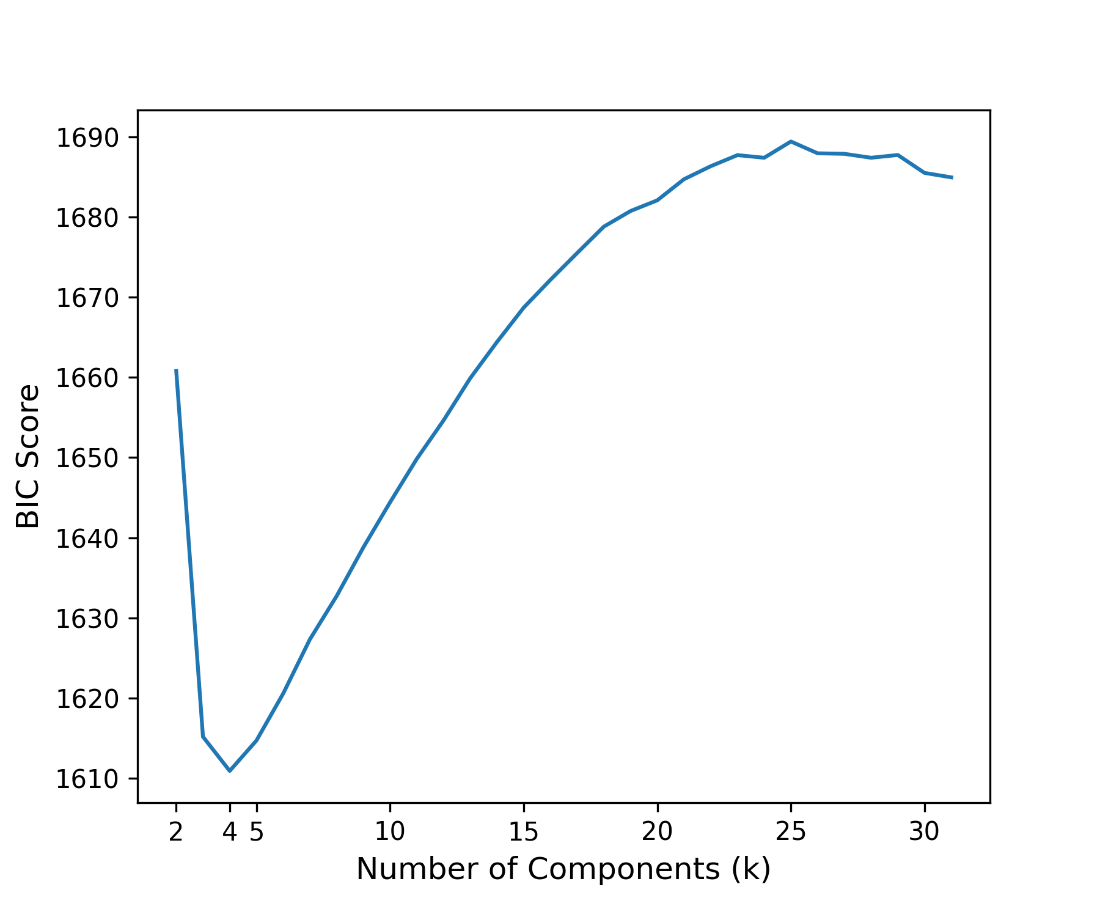


**Figure S4. The Averaged Bayesian Information Criterion (BIC scores) of wGMM models with different numbers of components.**

The wGMM models built for the torsion angle ϕ, with a series number of components ranging from 2 to 30 were evaluated on CASP12 FM dataset. The averaged BIC scores were calculated over all target proteins. wGMM models with 4 components achieved the lowest BIC score that balanced model complexity and accuracy well and were chosen in our study.


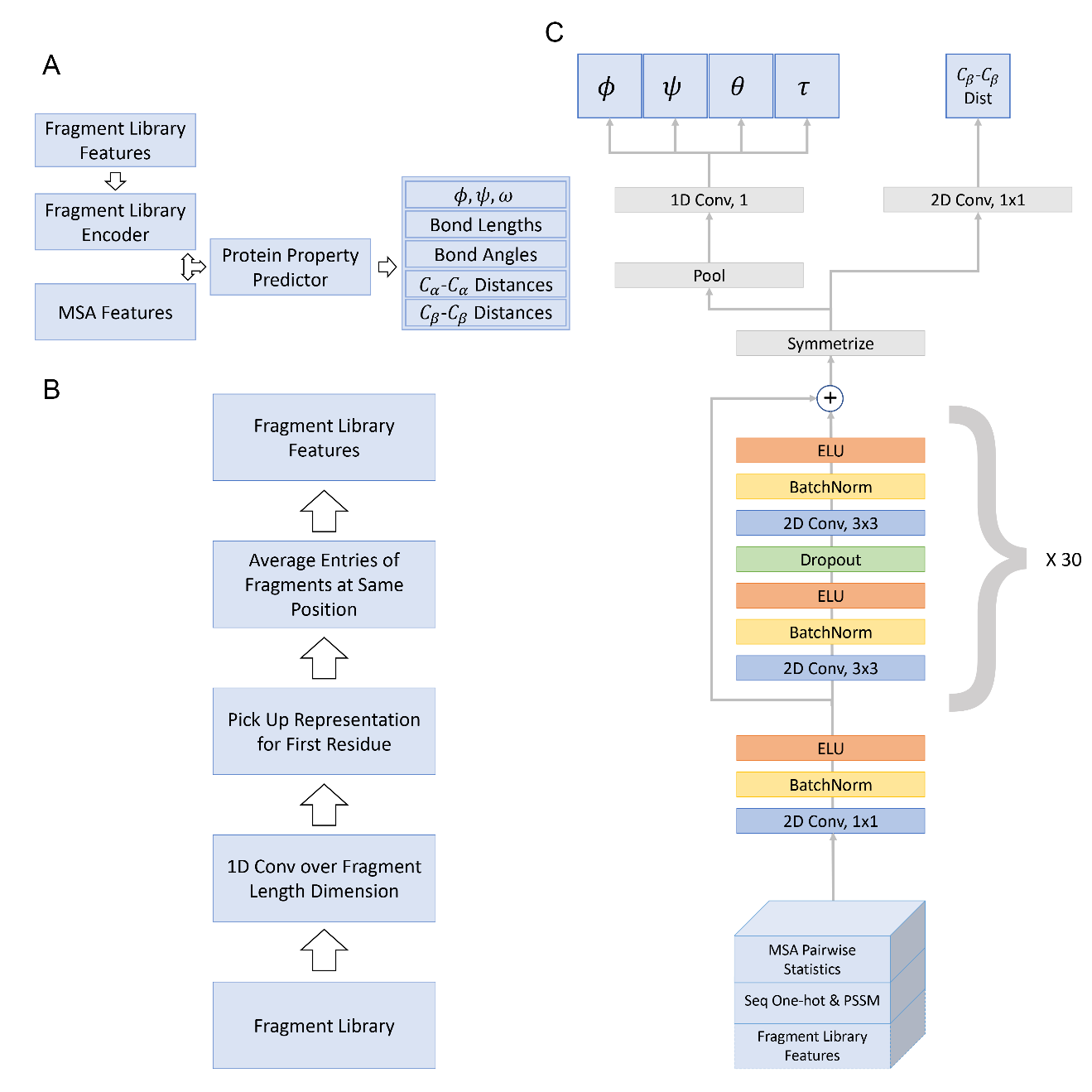


**Figure S5. The deep neural network using fragment libraries for protein property prediction.**

A: The overall pipeline of the deep neural network with a fragment library encoder and a protein property predictor. B: The pipeline of the fragment library encoder that transforms a raw fragment library into 1D features. C: The pipeline of the protein property predictor that takes the output of the fragment library encoder as well as sequential information as input and predicts multiple 1D and 2D structural properties.


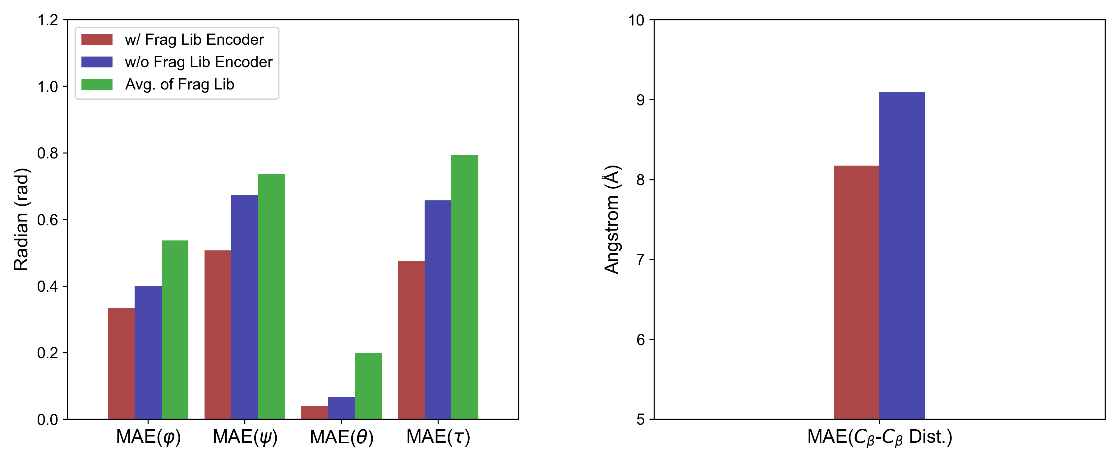


**Figure S6. Performance analysis of the deep neural network for protein property prediction, evaluated on the CASP13 FM test set.**

Related to Figure 3. The mean absolute error (MAE) of torsion angles and the MAE of C_β_-C_β_ pairwise distances are shown in the left and right subfigures, respectively.


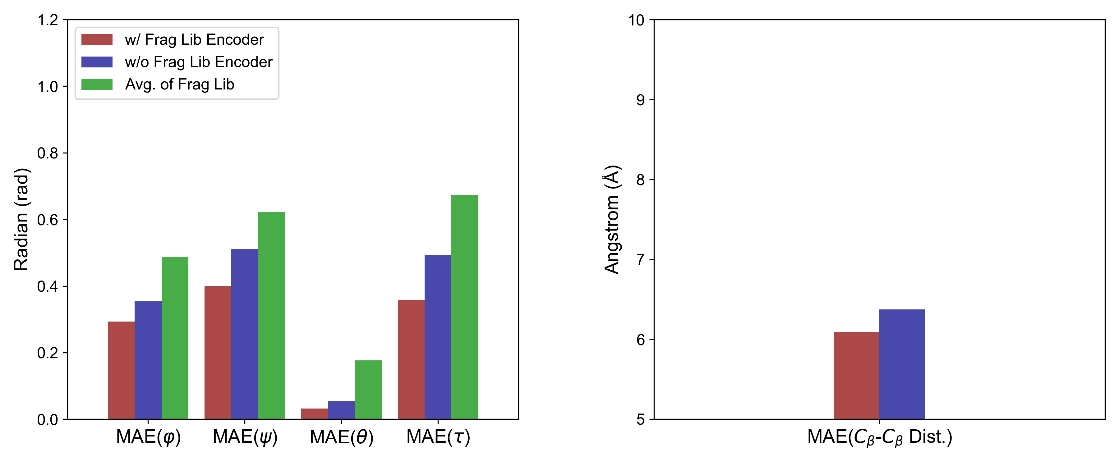


**Figure S7. Performance analysis of the deep neural network for protein property prediction, evaluated on the CASP13 TBM test set.**

The categories and evaluation metrics on each subfigure are the same as those in Figure S6.


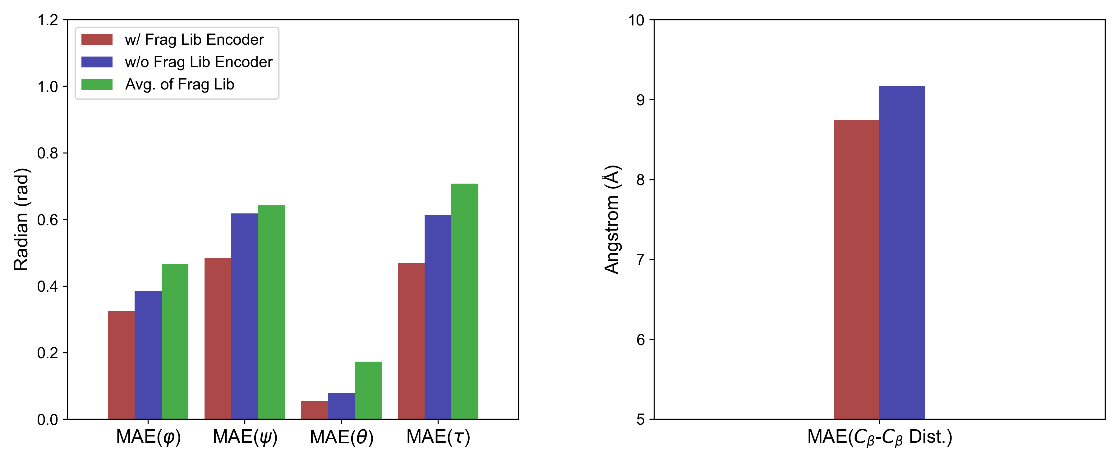


**Figure S8. Performance analysis of the deep neural network for protein property prediction, evaluated on the CAMEO test set.**

The categories and evaluation metrics on each subfigure are the same as those in Figure S6.


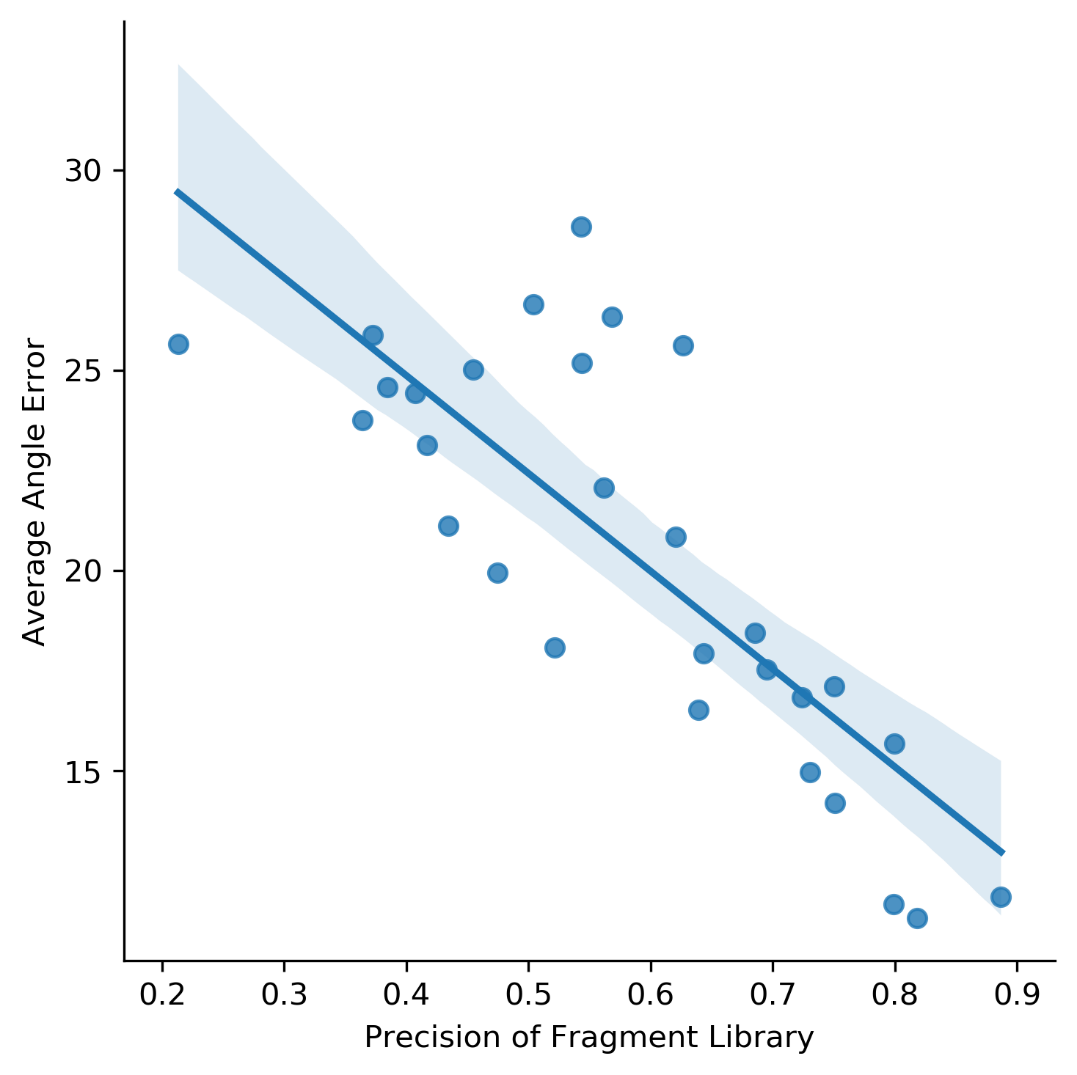


**Figure S9. Correlation between precisions of fragment library and averaged errors of torsion angles on CASP13 FM targets.**

The precision values had a strong negative correlation (ρ=-0.801) with the averaged errors of angles (ϕ and ψ) while two outliers (T0955-D1 and T1008-D1) were removed. This result indicates a high-quality fragment library can lead to more accurate predicted protein properties.

**Table S1. The performance of fragment-level structural properties of DeepFragLib on three independent test sets.**

| Test set | SSACC (%) | ERR_ϕ_ (°) | ERR_ψ_ (°) | ERR_θ_ (°) | ERR_τ_ (°) | ERR_Cα_ (Å) | ERR_Cβ_ (Å) |
| --- | --- | --- | --- | --- | --- | --- | --- |
| CASP13 FM | 90.470 | 30.728 | 42.237 | 11.352 | 45.422 | 0.716 | 1.066 |
| CASP13 FM* | 91.571 | 29.677 | 40.613 | 10.993 | 43.864 | 0.677 | 1.020 |
| CASP13 TBM | 89.870 | 27.912 | 35.655 | 10.108 | 38.614 | 0.564 | 0.901 |
| CASP13 TBM* | 90.491 | 26.575 | 34.138 | 9.747 | 37.168 | 0.522 | 0.846 |
| CAMEO | 91.190 | 26.752 | 36.805 | 9.901 | 40.535 | 0.613 | 0.947 |
| CAMEO* | 91.637 | 25.732 | 35.588 | 9.613 | 37.402 | 0.578 | 0.903 |

The SSACC, ERR_ϕ_, ERR_ψ_, ERR_θ_, ERR_τ_, ERR_Cα_and ERR_Cβ_ of fragment libraries constructed by DeepFragLib for CASP13 FM, CASP13 TBM and CAMEO are shown in this table. The rows marked with asterisks (*) show the performance of DeepFragLib with weighted fragments by predicted RMSD values (see Methods for details). The weighted fragment libraries achieved better performance on each kind of properties (i.e., the higher value of SSACC and the lower value of the remaining metrics) when compared with the vanilla fragment libraries constructed by DeepFragLib.

**Table S2. TM-Scores of decoys generated by Rosetta with/without DeepFragLib evaluated on CASP13 FM domains.**

| Category | Rosetta w/ DeepFragLib | | | Rosetta w/o DeepFragLib | | |
| --- | --- | --- | --- | --- | --- | --- |
| Number of decoys | 20 | 200 | 2000 | 20 | 200 | 2000 |
| Mean of TM-Score | 0.450 | 0.493 | 0.543 | 0.436 | 0.492 | 0.527 |
| Median of TM-Score | 0.416 | 0.472 | 0.532 | 0.403 | 0.454 | 0.489 |
| Targets with TM-Score > 0.5 | 9 | 14 | 17 | 6 | 12 | 14 |

For each group, different numbers of decoys were generated by Rosetta AbinitioRelax with fragment libraries constructed by DeepFragLib (shown as “Rosetta w/ DeepFragLib” groups) or with fragment libraries constructed by its default fragment library construction algorithm, NNMake (shown as “Rosetta w/o DeepFragLib” groups). The best decoy with the highest TM-Score for each domain target was picked respectively, and the mean, median and the number of best decoys with correct topology (TM-Score > 0.5) were evaluated. Rosetta with DeepFragLib generally outperforms that with NNMake, and the performance gap between the two algorithms grows larger with more predicted structures.

**Table S3. Domain targets in the CASP13 FM test set.**

| Target | Length |
| --- | --- |
| T0950-D1 | 342 |
| T0953s1-D1 | 67 |
| T0953s2-D1 | 44 |
| T0953s2-D3 | 77 |
| T0955-D1 | 41 |
| T0957s2-D1 | 155 |
| T0958-D1 | 77 |
| T0960-D2 | 84 |
| T0963-D2 | 82 |
| T0968s1-D1 | 118 |
| T0968s2-D1 | 115 |
| T0969-D1 | 354 |
| T0978-D1 | 413 |
| T0981-D3 | 203 |
| T0986s1-D1 | 92 |
| T0986s2-D1 | 155 |
| T0987-D1 | 185 |
| T0989-D1 | 134 |
| T0989-D2 | 112 |
| T0990-D1 | 76 |
| T0990-D3 | 213 |
| T0992-D1 | 107 |
| T0997-D1 | 185 |
| T0998-D1 | 166 |
| T1001-D1 | 139 |
| T1005-D1 | 326 |
| T1008-D1 | 77 |
| T1010-D1 | 210 |
| T1015s1-D1 | 88 |
| T1019s1-D1 | 58 |
| T1022s1-D1 | 156 |

**Table S4. Domain targets in the CASP13 TBM test set.**

| Target | Length | Target | Length |
| --- | --- | --- | --- |
| T0951-D1 | 266 | T0995-D1 | 294 |
| T0957s1-D2 | 54 | T0996-D1 | 107 |
| T0960-D3 | 89 | T0996-D2 | 127 |
| T0960-D5 | 105 | T0996-D3 | 100 |
| T0961-D1 | 503 | T0996-D4 | 133 |
| T0962-D1 | 177 | T0996-D5 | 121 |
| T0963-D3 | 93 | T0996-D6 | 104 |
| T0963-D5 | 94 | T0996-D7 | 140 |
| T0964-D1 | 95 | T0999-D2 | 453 |
| T0965-D1 | 313 | T0999-D3 | 180 |
| T0966-D1 | 492 | T0999-D4 | 244 |
| T0967-D1 | 79 | T0999-D5 | 288 |
| T0971-D1 | 130 | T1002-D1 | 59 |
| T0974s1-D1 | 69 | T1002-D2 | 59 |
| T0976-D1 | 120 | T1002-D3 | 144 |
| T0976-D2 | 124 | T1004-D1 | 86 |
| T0977-D1 | 301 | T1004-D2 | 77 |
| T0977-D2 | 204 | T1006-D1 | 77 |
| T0979-D1 | 92 | T1009-D1* | 718 |
| T0981-D1 | 86 | T1014-D2 | 117 |
| T0981-D4 | 111 | T1015s2-D1 | 129 |
| T0981-D5 | 127 | T1016-D1 | 202 |
| T0982-D1 | 135 | T1017s1-D1 | 110 |
| T0982-D2 | 132 | T1018-D1 | 334 |
| T0983-D1 | 236 | T1019s2-D1 | 88 |
| T0985-D1* | 842 | T1021s1-D1 | 149 |
| T0993s1-D1 | 263 | T1021s2-D1 | 349 |
| T0993s2-D1 | 98 | T1022s1-D2 | 67 |

Targets marked with star are those that were not evaluated in the quality analysis of fragment libraries since Flib-Coevo failed to construct fragment libraries for these targets.

**Table S5. Domain targets in the CAMEO test set.**

| Target | Length | Target | Length | Target | Length | Target | Length |
| --- | --- | --- | --- | --- | --- | --- | --- |
| 5OQKA | 147 | 6BZKA | 57 | 6GCJA | 121 | 6NFWA* | 151 |
| 5WB4H | 193 | 6BZTD | 501 | 6GDJB | 69 | 6NJYB | 237 |
| 5YA6B | 165 | 6CCIA | 354 | 6GF8B | 105 | 6NK8B | 204 |
| 5YVQB | 103 | 6CGHA | 89 | 6GHOB | 274 | 6NNBA | 59 |
| 5Z34A | 361 | 6CMKA | 375 | 6GMSA | 132 | 6NU4A | 56 |
| 5Z3FA | 371 | 6CP8B | 162 | 6GOCA | 444 | 6NWHB | 163 |
| 5Z3KB | 483 | 6CP8D | 157 | 6GW7A | 59 | 6NX4A | 167 |
| 5Z6DB | 204 | 6CP9G | 109 | 6GXRA | 360 | 6O0QB | 140 |
| 5Z8BB | 204 | 6CP9H | 114 | 6H1WB | 210 | 6O1QA | 119 |
| 5Z9TB | 499 | 6CZTA | 82 | 6H2XA | 352 | 6O2KB | 142 |
| 5ZB2A | 402 | 6D0IC | 159 | 6H3AB | 57 | 6OM5A | 252 |
| 5ZKEA | 174 | 6D0ID | 71 | 6H56B | 179 | 6OMPB | 356 |
| 5ZKTB | 55 | 6D2WB* | 685 | 6H6NB | 118 | 6OX6A | 194 |
| 5ZNSA | 381 | 6D2YA* | 428 | 6HLKA | 131 | 6OX6B | 76 |
| 5ZORA | 149 | 6D7YA* | 92 | 6HP1A | 297 | 6PDKA | 248 |
| 5ZT7B | 252 | 6D7YB | 155 | 6HPBC | 52 | 6PIEA | 162 |
| 5ZTCB | 197 | 6D97D | 522 | 6HQCA | 116 | 6PWDA | 176 |
| 5ZYOD | 152 | 6D9FB | 322 | 6HXAA | 375 | 6QBLA | 93 |
| 6A0EB | 304 | 6D9MA | 326 | 6I9HA | 92 | 6QEUA | 82 |
| 6A2UC | 114 | 6DAND | 323 | 6IL9A | 489 | 6R5WC | 169 |
| 6A2UD | 530 | 6DIIL* | 616 | 6J0YD* | 53 | 6R6MB | 100 |
| 6A2WA | 166 | 6DKAI | 226 | 6JIEA | 77 | 6RFGD | 123 |
| 6A4CA | 215 | 6DL4A | 119 | 6JILD | 298 | 6RJWA | 161 |
| 6A51B | 158 | 6DRFA | 143 | 6JO0A | 224 | 6RPQA | 249 |
| 6A5DB | 93 | 6E0KA | 295 | 6JPHA* | 770 | 6S5WB | 104 |
| 6A5ED | 84 | 6E1RF* | 544 | 6JQFA* | 734 | 6S5YH | 160 |
| 6A5FB | 151 | 6E3CC | 134 | 6JYZA | 455 | 6SGOA | 136 |
| 6A5GB | 157 | 6E6AA | 163 | 6KFNA | 298 | 6SJ8B | 305 |
| 6AEFA | 458 | 6E9MA | 90 | 6KZ6B | 147 | 6SX1A | 79 |
| 6AF3G | 117 | 6EDBB | 67 | 6L6GB | 264 | 6TD9B | 247 |
| 6AHQT | 107 | 6FP5B | 86 | 6MICA | 164 | 6UCHA | 41 |
| 6AHTA | 111 | 6FTOC | 75 | 6MLTA* | 603 | 6UKEX | 258 |
| 6BEAA | 433 | 6FXDB | 121 | 6MLYD* | 772 |  |  |
| 6BS5A | 330 | 6G7GA | 115 | 6MM1D | 135 |  |  |
| 6BZJA | 57 | 6G8YA | 92 | 6N63A | 141 |  |  |

Targets marked with star are those that were not evaluated in the quality analysis of fragment libraries since Flib-Coevo failed to construct fragment libraries for these targets.

| Target | Length |
| --- | --- |
| T0863-D1 | 193 |
| T0863-D2 | 356 |
| T0864-D1 | 246 |
| T0866-D1 | 104 |
| T0868-D1 | 116 |
| T0869-D1 | 104 |
| T0870-D1 | 123 |
| T0880-D1 | 36 |
| T0880-D2 | 157 |
| T0884-D1 | 71 |
| T0886-D2 | 127 |
| T0888-D1 | 121 |
| T0890-D1 | 82 |
| T0890-D2 | 106 |
| T0892-D1 | 69 |
| T0892-D2 | 110 |
| T0894-D1 | 89 |
| T0894-D2 | 54 |
| T0896-D1 | 86 |
| T0896-D3 | 161 |
| T0897-D1 | 138 |
| T0897-D2 | 124 |
| T0898-D1 | 106 |
| T0898-D2 | 55 |
| T0900-D1 | 102 |
| T0901-D1 | 223 |
| T0904-D1 | 251 |
| T0905-D1 | 242 |
| T0912-D3 | 103 |
| T0914-D2 | 162 |
| T0915-D1 | 154 |
| T0918-D1 | 108 |
| T0918-D2 | 123 |
| T0946-D1 | 80 |

**Table S6. Domain targets in the CASP12 FM set.**

**Table S7. Targets in the CASP14 FM test set.**

| Target | Length |
| --- | --- |
| T1029 | 126 |
| T1031 | 96 |
| T1033 | 101 |
| T1037 | 405 |
| T1038 | 191 |
| T1039 | 162 |
| T1040 | 131 |
| T1041 | 243 |
| T1042 | 277 |
| T1043 | 149 |
| T1074 | 133 |
| T1090 | 190 |

**Table S8. Average Lib956 MSA Depth of the test sets.**

|  | CASP13 FM | CASP13 TBM | CAMEO | CASP14 FM |
| --- | --- | --- | --- | --- |
| Avg. MSA Depth | 0.097 | 0.554 | 0.136 | 0.000 |

**Supplementary references**

1. Rohl CA, Strauss CE, Misura KM, Baker D. Protein structure prediction using Rosetta. Methods in enzymology. 383: Elsevier; 2004. p. 66-93.

2. Gront D, Kulp DW, Vernon RM, Strauss CE, Baker D. Generalized fragment picking in Rosetta: design, protocols and applications. PLoS One. 2011;6(8):e23294.

3. Wang T, Qiao Y, Ding W, Mao W, Zhou Y, Gong H. Improved fragment sampling for ab initio protein structure prediction using deep neural networks. Nat Mach Intell. 2019;1(8):347-55.

4. Zhang Y, Skolnick J. TM-align: a protein structure alignment algorithm based on the TM-score. Nucleic Acids Res. 2005;33(7):2302-9.
